# Anchoring NiO Nanosheet on the Surface of CNT to Enhance the Performance of a Li-O_2_ Battery

**DOI:** 10.3390/nano12142386

**Published:** 2022-07-13

**Authors:** Shuang Chen, Shukun Wang, Yunyun Dong, Hongmei Du, Jinsheng Zhao, Pengfang Zhang

**Affiliations:** 1 State Key Laboratory of Heavy Oil Processing, College of Chemical Engineering, China University of Petroleum (East China), Qingdao 266580, China; chsh1030@163.com (S.C.); wsk971008@163.com (S.W.); 2 Shandong Provincial Key Laboratory of Chemical Energy Storage and Novel Cell Technology, School of Chemistry and Chemical Engineering, Liaocheng University, Liaocheng 252059, China; dongyunyun@lcu.edu.cn (Y.D.); duhongmei@lcu.edu.cn (H.D.)

**Keywords:** Li-O_2_ battery, transition-metal oxides, porous NiO nanosheet, electrocatalyst

## Abstract

Li_2_O_2_, as the cathodic discharge product of aprotic Li-O_2_ batteries, is difficult to electrochemically decompose. Transition-metal oxides (TMOs) have been proven to play a critical role in promoting the formation and decomposition of Li_2_O_2_. Herein, a NiO/CNT catalyst was prepared by anchoring a NiO nanosheet on the surface of CNT. When using the NiO/CNT as a cathode catalyst, the Li-O_2_ battery had a lower overpotential of 1.2 V and could operate 81 cycles with a limited specific capacity of 1000 mA h g^−1^ at a current density of 100 mA g^−1^. In comparison, with CNT as a cathodic catalyst, the battery could achieve an overpotential of 1.64 V and a cycling stability of 66 cycles. The introduction of NiO effectively accelerated the generation and decomposition rate of Li_2_O_2_, further improving the battery performance. SEM and XRD characterizations confirmed that a Li_2_O_2_ film formed during the discharge process and could be fully electrochemical decomposed in the charge process. The internal network and nanoporous structure of the NiO/CNT catalyst could provide more oxygen diffusion channels and accelerate the decomposition rate of Li_2_O_2_. These merits led to the Li-O_2_ battery’s better performance.

## 1. Introduction

In the past few years, as the global population and economy increase rapidly, the development model, relying on non-renewable fossil fuels as the main energy source, has been unable to meet human needs. Researchers are devoted to seeking high-energy and clean energy storage facilities to replace non-renewable resources. With a super-high theory energy density of 3500 Wh kg^−1^, Li-O_2_ batteries are poised to become the next generation of rechargeable systems to store energy [1,2,3,4]. Particularly, in an aprotic Li-O_2_ battery system, lithium peroxide (Li_2_O_2_) is recognized as the main discharge product, and is generated via the oxygen reduction reaction (ORR) and its decomposition into Li^+^ ions and O_2_ through the oxygen evolution reaction (OER) during the recharge process [5,6]. The reaction equation is expressed as 2Li^+^ + 2e^−^ + O_2_ ↔ Li_2_O_2_ (E_0_ = 2.96 V vs. Li/Li^+^). So far, the actual energy density of Li-O_2_ batteries is far from its theoretical value. Additionally, reasons for this primarily include: (i) the inherently slow kinetics in ORR and OER throughout discharge and recharge processes can result in high overpotential and poor cyclic stability; (ii) the Li_2_O_2_ discharge products, generated on the surface of electrode material, occupy the active site; and (iii) the decomposition of organic electrolytes at higher voltages around 4.5 V can lead to increased impedance [7,8,9].

In this regard, to improve the formation and decomposition kinetics of Li_2_O_2_, the design and preparation of efficient catalysts for application to Li-O_2_ batteries is necessary. Up to now, a variety of cathode catalysts have been prepared and applied to Li-O_2_ batteries to reduce the overpotential of internal reactions [10,11,12]. Pt [13,14], Ru [15,16], Ir [17], Au [18] and Pd [19] have efficient catalytic activity for the formation and decomposition of Li_2_O_2_. However, scarce reserves and high prices have limited the practical application of noble-metal catalysts. TMOs such as Mn_2_O_3_ [20], Co_3_O_4_ [21,22,23] and CeO_2_ [24,25] show excellent cycle stability. Their features, such as a low cost, high activity and long-term stability, have made TMO one of the most promising candidates. During the discharge process, O_2_ is reduced and the insoluble discharge product Li_2_O_2_ forms. As a result, the design of catalysts that can provide enough space for storing Li_2_O_2_ is indispensable.

Carbon materials, considered as common electrode materials, possess excellent properties, including environmental friendliness, brilliant electrical conductivity and a high surface area, which have been paid a lot of attention. In Li-O_2_ batteries, carbon materials exhibit prominent catalytic performance as electrocatalysts owing to the complex porous structure [26,27]. They are usually considered as important carriers in cathode catalysts [28,29,30,31,32]. NiO-based catalysts, due to their high catalytic activity, have often been compounded with other chemical compounds such as metal oxides/sulfides [33,34,35,36,37]. The Li-O_2_ battery, with these NiO-based catalysts, shows a better cycling stability. However, the catalytic behavior of individual NiO catalysts is still ambiguous and should be further investigated. In particular, Zhao et al. introduced a NiO/MWCNT nanocomposite into Li-O_2_ batteries to study its electrochemical performance [38]. Their research revealed that Li-O_2_ batteries with a NiO/MWCNT catalyst exhibit a discharge capacity of 2500 mA h g^−1^ and a cycling ability of five cycles.

Herein, pristine CNT (CNT-P) was treated with concentrated acid to generate an oxygen-containing functional group, and then a NiO nanosheet was anchored on the surface of CNT to synthesize the NiO/CNT electrocatalyst. A Li-O_2_ battery with a NiO/CNT cathodic catalyst was assembled to study the catalytic performance for the formation and decomposition of Li_2_O_2_ and the cycling stability of batteries. With the NiO/CNT catalyst, the Li-O_2_ battery could sustain a stability of 81 cycles and an overpotential of 1.2 V with a constant capacity of 1000 mA h g^−1^ at a current density of 100 mA g^−1^. SEM and XRD analyses of the discharge cathodes show that solid Li_2_O_2_ adhered to the surface of both NiO/CNT during the discharge process. After recharging, the surface of NiO/CNT was clean, confirming that NiO/CNT could effectively catalyze the decomposition of Li_2_O_2_.

## 2. Experimental Section

### 2.1. Acidification of CNT

Based on the previous literature [31], 100 mL of concentrated nitric acid and concentrated sulfuric acid (*v*:*v* = 1:1) was sequentially added dropwise to 500 mg carbon nanotubes (Beijing Boyu Gaoke New Material Technology Co., Ltd. (Beijing, China)); diameter: 10–30 nm; purity: 95%). The suspension was then evenly dispersed by ultrasonication for two hours. After that, the mixtures were refluxed at 60 °C for eight hours. Then, the mixture was centrifuged and then washed with deionized water until the pH of the solution reached 7. Finally, the obtained product was dried overnight at 80 °C.

### 2.2. Preparation of NiO/CNT

A total of 100 mg of activated CNT and 1 mmol of NiCl_2_·6H_2_O were added into 70 mL of deionized water, and were well-dispersed by ultrasonication for 30 min. Then, NH_3_·H_2_O was added to the mixture dropwise until the pH value was equal to 11. Subsequently, the mixture was stirred for one hour and was transferred to a Teflon high-pressure reactor. Then, the temperature of the reaction equipment was increased to 180 °C and maintained for 12 h. The mixture was washed with deionized water and dried at 80 °C. The obtained solid product was calcined by tube furnace at 400 °C for 3 h under N_2_ atmosphere at the ramp rate of 2 °C min^−1^.

### 2.3. Material Characterization

Scanning electron microscopy (SEM) images of the materials were recorded on Thermo Fisher Scientific FIB-SEM GX4, operating at 10 kV. Transmission electron microscopy (TEM) and high-resolution transmission electron microscope (HRTEM) of the materials were carried out on JEM-2100 using ethanol absolute as the dispersant. Powder X-ray diffraction (PXRD) pattern was carried out on a Kigaka D/max 2500 X-ray advance diffractometer with a monochromatic Cu Kα radiation source (λ = 1.54 Å, 10° min^−1^ from 20° to 80°). The Raman spectra of the materials were recorded on a Renishaw Laser Microscopic Confocal Raman Spectrometer accompanied by excitation wavelength of 532 nm. X-ray photoelectron spectroscopy (XPS) was examined using Thermo Fisher Scientific ESCALAB Xi+ spectrometer (Thermo Fisher Scientific Inc., Waltham, MA, USA). Nitrogen adsorption–desorption isotherm was investigated with ASAP 2460-3 (Micromeritics) multi-station extended specific surface area and porosity analyzer at 77.3 K.

### 2.4. Electrochemical Tests

First, the as-prepared catalyst was mixed with polyvinylidene fluoride (PVDF, 10 wt%) in 600 μL of N-methylpyrrolidone (NMP) at a mass ratio of 9:1. The mixture was ball-milled for three hours to obtain a uniform slurry. After that, the slurry was dripped onto the carbon paper (TGP-H-060, Toray Corporation, Tokyo, Japan) with a diameter of 7 mm. The load mass of the electrode sheet was approximately 0.5 mg cm^−2^. Then, carbon paper was dried under vacuum with a temperature of 100 °C for 10 h. The battery was assembled under an Ar atmosphere while O_2_ and H_2_O content were kept below 0.1 ppm. Additionally, a CR 2032-type coin cell was made of positive cap with seven holes (Φ = 2 mm), lithium foil, glass fiber separators (Whatman A), electrolyte (1 M LITFSI/TEGDME), spacer, spring and negative cap. Oxygen was then pumped into the closed container with the battery. After standing for three hours, the assembled batteries were tested on the LAND CT2001A with the galvanostatic charge–discharge testing to determine their cycling performance. The cyclic voltammetry (CV) curve was tested with electrochemical workstation (PGSTAT 302N, Metrohm) with the voltage range of 2.0–4.5 V, and the sweep rate was set to 0.1 mV s^−1^. Kinetics and electrode interface structure information were obtained using electrochemical impedance spectroscopy (EIS). The frequency varied between 100 kHz and 0.1 Hz.

## 3. Results and Discussion

### 3.1. Characterizations of Materials’ Morphology and Structure

**Figure 1:**
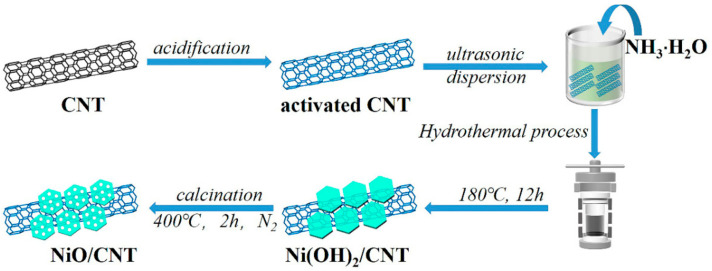
Schematic illustration of the fabrication of NiO/CNT composites.

**Figure 2:**
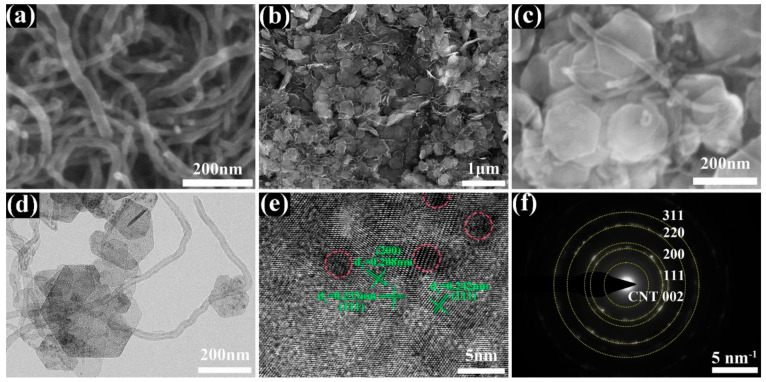
(**a**–**c**) SEM images of CNT and NiO/CNT, (**d**) TEM, (**e**) HRTEM images, (**f**) SAED pattern of NiO/CNT.

As shown in Figure 1, the NiO/CNT electrocatalyst was synthesized using the facile hydrothermal process, followed by calcining at a certain temperature. First, NH_3_·H_2_O, as a precipitant, was combined with Ni^2+^ to form a Ni(OH)_2_ precursor. Then, the precursor was further crystallized through the process of hydrothermal reaction. The morphology of the materials was observed by SEM. The XRD patterns of Ni(OH)_2_ and Ni(OH)_2_/CNT are displayed in Figure S1a. The main diffraction peaks at 19.1°, 33.0°, 38.5°, 51.9° and 59.0°, 59.9° are well-matched with the (001), (100), (011), (012), (100) and (003) planes of Ni (OH)_2_ (JCPDS #00-014-0117), respectively, demonstrating the formation of Ni (OH)_2_ [39]. A hexagonal structure was seen in the Ni (OH)_2_ sample (Figure S1b). As is shown in Figure 2a, CNTs were criss-crossed and woven from tubular structures with a diameter of about 25 nm. Through the subsequent calcination process, Ni(OH)_2_ released gaseous water molecules at a high temperature to form porous NiO, and the hexagonal morphology was maintained. Figure 2b,c show the morphology of NiO/CNT. It can be seen that NiO and CNT were uniformly compounded together. According to HRSEM images, it can be observed that CNTs were evenly and tightly coated on the surface of NiO nanosheets. The fine structure of NiO/CNT can be observed in TEM and HRTEM images. CNT and the porous NiO nanosheet were closely linked with each other (Figure 2d). Furthermore, it was apparent that the porous structure was uniformly distributed on the NiO nanosheet. Furthermore, as exhibited in Figure 2e, the lattice fringe spacings of 0.24 nm were well-indexed to the (111) crystal plane of NiO. Furthermore, the diffraction rings in the selected area electron diffraction (SAED) pattern were indexed into the (002) plane of CNT and the (111), (200), (220) and (311) planes of NiO (Figure 2f).

**Figure 3:**
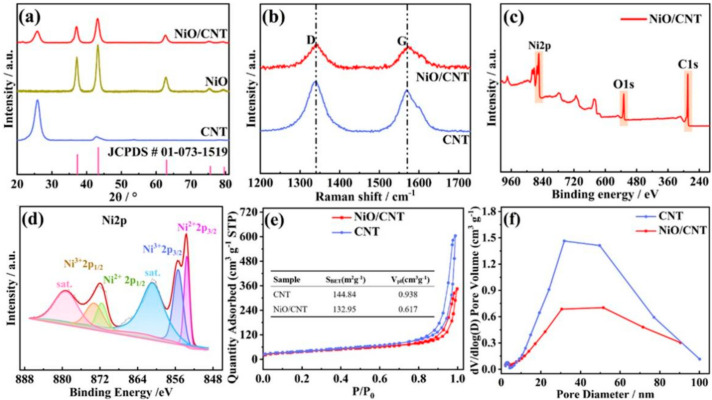
(**a**) XRD patterns of NiO/CNT, NiO and CNT catalysts; (**b**) Raman spectra of NiO/CNT and CNT catalysts; (**c**) XPS survey spectrum; (**d**) Ni 2p high-resolution spectrum of NiO/CNT; (**e**) N_2_ adsorption–desorption isotherms; and (**f**) pore diameter distributions of NiO/CNT and CNT.

XRD patterns of activated CNT were performed, and the results can be seen in Figure 3a. It is obvious that there were two peaks near 26° and 43° in the CNT XRD pattern, belonging to the diffraction peaks of the (005) and (102) planes. The XRD pattern of NiO/CNT exhibited five peaks at 37.2°, 43.2°, 62.8°, 75.4° and 79.4°, which were indexed to the (111), (200), (220), (311) and (222) planes of NiO, respectively, corresponding to the standard card PDF No. 01-073-1519. After that, Raman spectroscopy was carried out to further analyze the structure of the NiO/CNT catalyst, which revealed the graphite degree of NiO/CNT and CNT (Figure 3b). Obviously, the D-band representing the lattice defect of C atoms was around 1300 cm^−1^. Furthermore, the peak around 1580 cm^−1^ represents the G-band, which indicates as in-plane stretching vibration of the sp2 hybridization of C atoms [40]. According to the calculation, the I_D_/I_G_ value of NiO/CNT was 1.09, which is lower than the 1.2 of CNT. After loading Ni(OH)_2_ on the CNT, the Ni(OH)_2_/CNT was calcinated at 400 °C in the N_2_ atmosphere to prepare NiO/CNT. The calcination process led to the ratio decrease in the functional group on the surface of the CNT, resulting in the decrease in the ID/IG value. The lower the I_D_/I_G_ value, the more complete the surface structure of the carbon nanotubes, and the stronger electronic conduction. After compounding NiO nanosheets, the ordered structure of the carbon nanotube surface was improved, which facilitated the conduction of electrons between CNTs and NiO. Considering the perspective of battery performance, although there were fewer defects on the CNT section for NiO/CNT, the porous structure of NiO nanosheets provided more defects in composite materials, and so the cathode catalyst had an efficient catalytic ability for ORR and OER processes.

The element composition as well as valence distribution of NiO/CNT were further confirmed via XPS characterization. The C1s spectra are compared in Figure S2. The ratio of the C-O bond in NiO/CNT was about 17.7%, which is lower than the 20.2% seen in CNT. The calcination process at 400 °C in the N_2_ atmosphere to prepare the NiO/CNT catalyst may have led to the ratio decrease in the C-O bond. In addition, after loading the NiO on CNT, a new peak appeared, which should be ascribed to the Ni-O bond based on the O1s XPS spectra (Figure S3). Figure 3c exhibits the survey spectrum of NiO/CNT. The characteristic peaks of C, O and Ni elements were easily observed from the spectrum, proving the existence of these three elements. Moreover, the high-resolution spectrum of Ni 2p in NiO/CNT is displayed (Figure 3d). The two peaks situated at 855.5 and 871.6 eV belong to Ni^2+^. The peaks at 853.8 and 873.2 eV matched with Ni^3+^. The satellite peaks were well-fitted, abbreviated as ‘sat.’ [34,35]. According to the XPS data, it can be clearly seen that Ni existed on the surface of NiO/CNT, and mainly shows the valence state of Ni^2+^ and Ni^3+^. Thermogravimetry (TG) confirmed that the mass ratio of NiO in the NiO/CNT catalyst was 56.2% (Figure S4).

To obtain the specific surface area and pore size distribution of NiO/CNT and CNT catalysts, a nitrogen absorption and desorption measurement was carried out. As depicted in Figure 3e, nitrogen adsorption–desorption isotherms of CNT and NiO/CNT catalysts showed obvious hysteresis loops, confirming the existence of distinct mesopores [41,42]. These isotherms should be classified as type IV isotherms. Moreover, an obvious H3-type hysteresis loop was observed. According to Brunner−Emmet−Teller (BET) characterization, the specific surface area of CNT was 144.8 m^2^ g^−1^ and the pore volume was 0.938 cm^3^ g^−1^. When loading the NiO nanosheet on CNT, NiO/CNT could still achieve a specific surface area of 132.9 m^2^ g^−1^ and a pore volume of 0.617 cm^3^ g^−1^, which is slightly lower than that of CNT, confirming the porous inner structure. As shown in Figure 3f, a pore diameter of 30 nm can be observed based on the Barrett–Joyner–Halenda (BJH) calculation method, which comes from the interstitial holes formed by cross-linked CNTs [43]. The mesoporous structure of CNT could effectively store the Li_2_O_2_ products, which is beneficial for the improvement of the Li-O_2_ battery performance.

### 3.2. Electrochemical Performance Test

**Figure 4:**
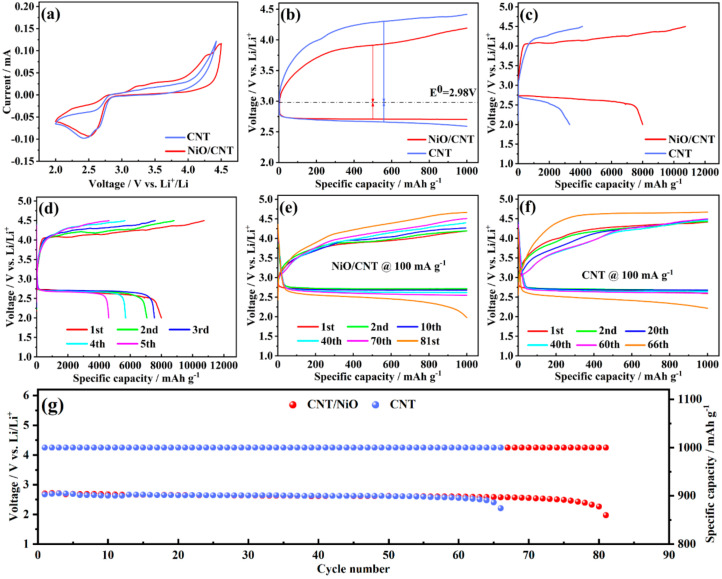
(**a**) The CV curves and (**b**) discharge–charge curves at first cycle of Li-O_2_ batteries with NiO/CNT and CNT catalysts; (**c**) full discharge–charge curves of NiO/CNT and CNT catalysts at a current density of 100 mA g^−1^; (**d**) the cycling stability of full discharge–charge battery with the NiO/CNT catalyst at a current density of 100 mA g^−1^; (**e**,**f**) the cycling curves of NiO/CNT and CNT catalysts with a limited specific capacity of 1000 mA h g^−1^ at a current density of 100 mA g^−^^1^; (**g**) cycling stability of Li-O_2_ batteries with NiO/CNT and CNT cathodes at a current density of 100 mA g^−1^.

The Li-O_2_ battery was assembled, and a variety of electrochemical tests were performed to gain insight into the catalytic activity of the NiO/CNT and CNT catalysts (Figure 4). Cycle voltammetry (CV) curves were performed to research the onset potential of ORR and OER by using NiO/CNT and CNT as cathodes. The scan range was set from 2.0 V to 4.5 V (Figure 4a). It can be clearly seen that there was an obvious reduction peak in the batteries with both NiO/CNT and CNT cathodes. The reduction peak of the Li-O_2_ battery with the NiO/CNT catalyst appeared at 2.52 V, while the reduction peak of CNT appeared at 2.42 V, indicating that the NiO/CNT catalyst had better ORR catalytic activity. In the positive scanning process, an oxidation peak was generated at 3.20 V in the battery with the NiO/CNT catalyst, while an unapparent peak at a higher position, 3.4 V, appeared with the CNT catalyst, confirming the better OER activity of the NiO/CNT catalyst. Therefore, a higher discharge plateau and lower charge plateau confirm the superior catalytic performance of NiO/CNT electrode materials, which can decrease overpotential and increase cycle stability.

The charge–discharge curves of the first cycle were used to compare the overpotential of two materials under a limited capacity at 1000 mA h g^−1^. The Li-O_2_ battery with the NiO/CNT cathode exhibited an obvious lesser overpotential of 1.2 V, while the overpotential with the CNT cathode was 1.64 V (Figure 4b). The overpotential of OER decreased from 1.32 V to 0.93 V, and the overpotential of ORR decreased from 0.32 V to 0.27 V. Therefore, NiO/CNT had higher activity than CNT in ORR and OER processes. Additionally, using NiO/CNT and CNT catalysts as cathode materials, full discharge–charge tests in the first cycle of batteries were performed with a current density of 100 mA g^−1^ (Figure 4c). In the discharge stage, the battery with the NiO/CNT cathode catalyst showed a specific capacity of 8000 mA h g^−1^, while that of the battery with CNT was merely 3300 mA h g^−1^. The high capacity is mainly ascribed to the catalyst’s prominent catalytic capability for accelerating the generation of Li_2_O_2_. The charging voltage of the Li-O_2_ battery with the NiO/CNT cathode reached to 4.32 V, which is lower than the 4.42 V achieved with the CNT cathode at a current density of 100 mA g^−1^. Upon reaching the set upper limit (4.5 V), the charging specific capacity visibly exhibited a higher value of 11,000 mA h g^−1^ with overcharging. Electrolyte decomposition is considered to be the main reason for this result. Therefore, it can be revealed that the NiO/CNT electrocatalyst exhibited a better OER performance than the CNT electrocatalyst. In addition, the discharge–charge curve of Li-O_2_ batteries with NiO/CNT was also tested with a discharge specific capacity of 6810 mA h g^−1^, indicating NiO/CNT electrocatalysts possess a most excellent catalytic performance among these electrode materials (Figure S5). Moreover, the Li-O_2_ battery with the NiO/CNT cathode lasted for five cycles in the full discharge and charge process (Figure 4d). It was found that though the specific capacity of the battery showed a downward trend, the battery with the NiO/CNT cathode exhibited a stable cyclability and a higher capacity, at over 4000 mA h g^−1^, after five cycles. A detailed data comparison is shown in Figure S6. To provide insight into the importance of the interfacial interaction between NiO and CNT in the NiO/CNT catalyst, the NiO/CNT-P catalyst was prepared by loading NiO on CNT-P without acid-etching treatment. When using NiO/CNT-P as cathodic catalysts, the Li-O_2_ battery showed an overpotential of 1.3 V, which is higher than the 1.2 V achieved with the NiO/CNT catalyst (Figure S7). This result proves that the strong interfacial interactions between NiO and CNT are responsible for the superior catalytic activity of the NiO/CNT catalyst.

To further investigate the influence of NiO content on the performance of Li-O_2_ batteries, a NiO/CNT catalyst with 37.3 wt% and 72.9 wt% NiO loading was also prepared (Figure S8). Additionally, the Li-O_2_ battery with NiO(56.2 wt%)/CNT showed an overpotential of 1.2 V, which is lower than the 1.33 V achieved with the NiO(72.9 wt%)/CNT catalyst and 1.42 V with the NiO(37.3 wt%)/CNT catalyst. This indicates the better catalytic activity of NiO(56.2 wt%)/CNT catalyst for the formation and decomposition of Li_2_O_2_.

Additionally, to compare the cycle performance of the two materials, galvanostatic charge–discharge tests were carried out with the specific capacity fixed at 1000 mA h g^−1^. As depicted in Figure 4e,f, the battery with the NiO/CNT cathode could achieve a stability of 81 cycles with low polarization at a current density of 100 mA g^−1^, and the battery with the CNT cathode could only run for 66 cycles (Figure 4g). Obviously, the battery with the NiO/CNT cathode exhibited a superior catalytic performance and better cycle stability than that with CNT cathode. The remarkable cycling stability of Li-O_2_ batteries with NiO/CNT catalysts is attributed to the superior catalytic activity of NiO/CNT towards the continuous formation and decomposition of Li_2_O_2_. The high specific surface area of CNT is expected to show excellent absorption ability towards the gas of O_2_, and the mesoporous structure could provide a large space for the deposition of Li_2_O_2_. The NiO nanosheet could effectively decrease the decomposition potential of Li_2_O_2_. The synergetic effect between NiO and CNT ensured the Li-O_2_ battery had an optimal performance.

### 3.3. Characterizations of Discharge Products

**Figure 5:**
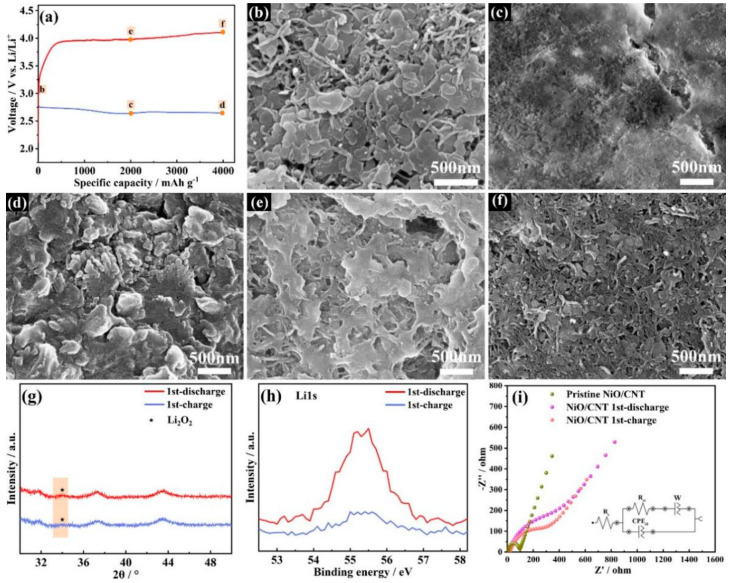
(**a**) The curves and SEM images of the CNT/NiO catalyst at different discharge–recharge depths at a current density of 100 mA g^−1^ discharged (blue line) to (**b**) the initial stage, (**c**) 2000 mA h g^−1^ (**d**) and 4000 mA h g^−1^, and recharged (red line) to (**e**) 2000 mA h g^−1^ and (**f**) 4000 mA h g^−1^; (**g**) XRD patterns and (**h**) Li 1s core peaks of the NiO/CNT catalyst after discharge and charge; (**i**) EIS of Li-O_2_ battery with NiO/CNT catalyst at different stages.

The discharge products were tested with SEM, XRD, XPS and EIS characterizations to better understand the charging and discharging mechanism of Li-O_2_ batteries. The different discharge–charge depths of the battery with the NiO/CNT catalyst were studied using SEM to analyze the morphology evolution of Li_2_O_2_ (Figure 5a). The NiO/CNT catalyst was clearly observed in the initial stage (Figure 5b). As the discharge depth reached 2000 mA h g^−1^, Li_2_O_2_ particles were deposited on the surface of NiO/CNT (Figure 5c). As the discharge depth increased to 4000 mA h g^−1^, Li_2_O_2_ gradually became thick (Figure 5d). When the charge depth was 2000 mA h g^−1^, only part of Li_2_O_2_ was decomposed (Figure 5e). Additionally, most of the discharge products disappeared when charge depth was 4000 mA h g^−1^ (Figure 5f).

To further corroborate the discharge product’s identity, the XRD of NiO/CNT electrode sheets after discharge and recharge processes were tested. As is shown in Figure 5g, the small peak in the yellow area corresponded to Li_2_O_2_. The result verifies that Li_2_O_2_ was indeed the primary discharge product of the Li-O_2_ battery. After recharging in the first cycle, the Li_2_O_2_ discharge product was almost completely decomposed, which illustrates that the NiO/CNT cathode had a high-efficiency catalytic ability to form and decompose Li_2_O_2_.

Additionally, the discharge product of the NiO/CNT cathode was further verified through XPS characterization under a capacity of 1000 mA h g^−1^. As seen in the Li 1s high-resolution spectra of the discharged NiO/CNT cathode, the peak at 55.2 eV corresponds to Li_2_O_2_ (Figure 5h). This result is in accord with the ex situ XRD data. After charging for the first cycle, the signal of the Li element in Li_2_O_2_ went almost completely undetected in the Li 1s high-resolution spectra, indicating that the battery with the NiO/CNT cathode had high reversibility throughout discharge–charge processes.

In addition, electrochemical impedance spectroscopy (EIS) was tested with a 1 M LiTFSI/TEGDME electrolyte to reflect the kinetic characteristics of charge transfer in Li-O_2_ batteries with a NiO/CNT electrocatalyst. In particular, the diameter of the semicircle in the mid-frequency range corresponds to the charge transfer resistance (R_ct_), which plays a vital role in determining the electrocatalytic capability and reaction kinetics. As shown in Figure S9 and Tables S1 and S2, the R_ct_ values of Li-O_2_ batteries with NiO/CNT and CNT catalysts were 84 Ω and 111 Ω in the initial stage, respectively. After discharge processes, the R_ct_ values increased to 414 Ω and 430 Ω, which decreased to 296 Ω and 310 Ω after the charge process. Owing to the formation of an insulating Li_2_O_2_, the impedance increased significantly after the discharge process. The result indicates that the NiO/CNT catalyst had a better electron conductivity, which is attributed to the better affinity between CNT and NiO nanosheets. In addition, the lower resistance after discharge processes should be ascribed to the good contact of the interface between the NiO/CNT catalyst and Li_2_O_2_, which benefits the decomposition of Li_2_O_2_ in the charge process. Therefore, after the charge process, the R_ct_ value of the Li-O_2_ battery with the NiO/CNT catalyst was lower than that with the CNT catalyst. The lower charge transfer resistance could ensure the better battery performance.

To explore the decay mechanism of the Li-O_2_ battery, the failed battery was disassembled. As depicted in Figure S10a,b the digital photos show that the electrolyte was almost consumed, and the surface of the Li anode was broken. These are likely the main factors behind the decay of batteries. The Li-O_2_ battery is an open system that will lead to the volatilization of the electrolyte. The rebuilt battery with the cycled NiO/CNT cathode, fresh electrolyte and a new Li anode demonstrated a decreased overpotential, reaffirming the decay mechanism (Figure S10c). Thus, the degradation of the Li anode and the loss of the electrolyte are the main reasons for the battery degradation.

## 4. Conclusions

A NiO/CNT catalyst was successfully prepared by anchoring a NiO nanosheet on the surface of CNT as an efficient electrocatalyst in a non-aqueous Li–O_2_ battery. NiO and CNT were uniformly compounded together to form a porous network structure during the hydrothermal process. Charge transfer dynamics were enhanced with the internal network structure in the CNT/NiO catalyst, and more oxygen transmission channels were provided by its nanoporous structure, enabling the Li-O_2_ battery to have an excellent cycle stability. The battery with the NiO/CNT cathode exhibited an overpotential of 1.2 V and could maintain 81 cycles at a fixed capacity of 1000 mA h g^−1^ at the current density of 100 mA g^−1^. In comparison, when using CNT as a cathodic catalyst, the battery could achieve a cycling stability of 66 cycles with an overpotential of 1.64 V. The battery with the NiO/CNT cathode possessed a specific discharge capacity of 8000 mA h g^−1^, while the specific capacity of the battery with the CNT cathode was merely 3300 mA h g^−1^. Additionally, SEM characterization verified that the Li_2_O_2_ particle generated in the discharge stage and completely electrochemically decomposed after recharging. XRD and XPS proved the formation and decomposition of Li_2_O_2_ as well. With the introduction of NiO, the catalyst can effectively catalyze the formation and decomposition of Li_2_O_2_ due to the high activity of the NiO/CNT electrocatalyst. This work puts forward an excellent design of a high-activity catalyst for a metal–O_2_ battery.

## Data Availability

The data presented in this study are available upon reasonable request from the corresponding author.

## Supplementary Materials

The following supporting information can be downloaded at: https://www.mdpi.com/article/10.3390/nano12142386/s1, Figure S1: (a) XRD patterns of Ni(OH)_2_/CNT and Ni(OH)_2_; (b) SEM image of Ni(OH)_2_/CNT; Figure S2: The C1s XPS spectra of CNT (a); NiO/CNT (b); the ratios of C element at different valence states (c); Figure S3: (a) The O1s XPS spectra of CNT and (b) NiO/CNT; Figure S4: The TG curve of NiO/CNT catalysts; Figure S5: Discharge/charge curve at first cycle of Li-O_2_ batteries with NiO/CNT. The NiO/CNT-mech electrode sheets are obtained by mixing CNT, NiO and PVDF with NMP solvent via ball-milling for 3 h; Figure S6: Five cycles of charge-discharge specific capacity comparison histogram of Li-O_2_ battery with NiO/CNT cathode; Figure S7: The charge and discharge curves of Li-O_2_ batteries with NiO/CNT and NiO/CNT-P catalysts; Figure S8: (a) The TG curve of NiO/CNT catalysts with different NiO contents (O_2_ atmosphere, 10 °C min^−1^); (b) the discharge and charge curves of Li-O_2_ battery with NiO/CNT catalysts at a current density of 100 mA g^−1^; Figure S9: EIS of pristine Li-O_2_ battery (blue), Li-O_2_ battery for the 1^st^-discharge (purple), Li-O_2_ battery for the 1^st^-charge (red); Figure S10: The photographs of (a) glass fiber and (b) lithium anode of Li-O_2_ battery after 81 cycles with the NiO/CNT catalyst; (c) The discharge-charge profiles between the battery at 81st cycle and the rebuilt battery with the cycled NiO/CNT cathode, the fresh lithium foil, glassfiber and electrolyte; Table S1: Parameters for the Li-O_2_ battery with the NiO/CNT catalyst, fitted by the equivalent circuit in the inset; Table S2: Parameters for Li-O_2_ battery with the CNT catalyst, fitted by the equivalent circuit in the inset.
