# Supplementary material for: Anchoring NiO Nanosheet on the Surface of CNT to Enhance the Performance of a Li-O2 Battery
Source: Nanomaterials (Basel). 2022 Jul 13;12(14):2386. doi: 10.3390/nano12142386 (PMC9320305; doi:10.3390/nano12142386)
Supplement: Supplementary file 1 [file nanomaterials-12-02386-s001.zip › nanomaterials-1788937-supplementary.pdf]

nanomaterials

*Supporting Information*

# Anchoring NiO Nanosheet on the Surface of CNT to Enhance the Performance of a Li-$O_2$ Battery

**Shuang Chen [1], Shukun Wang [1], Yunyun Dong [2], Hongmei Du [2], Jinsheng Zhao [2,*] and Pengfang Zhang [2,*]**

[1] State Key Laboratory of Heavy Oil Processing, College of Chemical Engineering, China University of Petroleum (East China), QingDao, 266580, China; chsh1030@163.com (S.C.); wsk971008@163.com (S.W.)
[2] Shandong Provincial Key Laboratory of Chemical Energy Storage and Novel Cell Technology, School of Chemistry and Chemical Engineering, Liaocheng University, Liaocheng 252059, China; dong-yunyun@lcu.edu.cn (Y.D.); duhongmei@lcu.edu.cn (H.D.)
* Correspondence: j.s.zhao@163.com (J.Z.); zhangpengfang111@163.com (P.Z.)

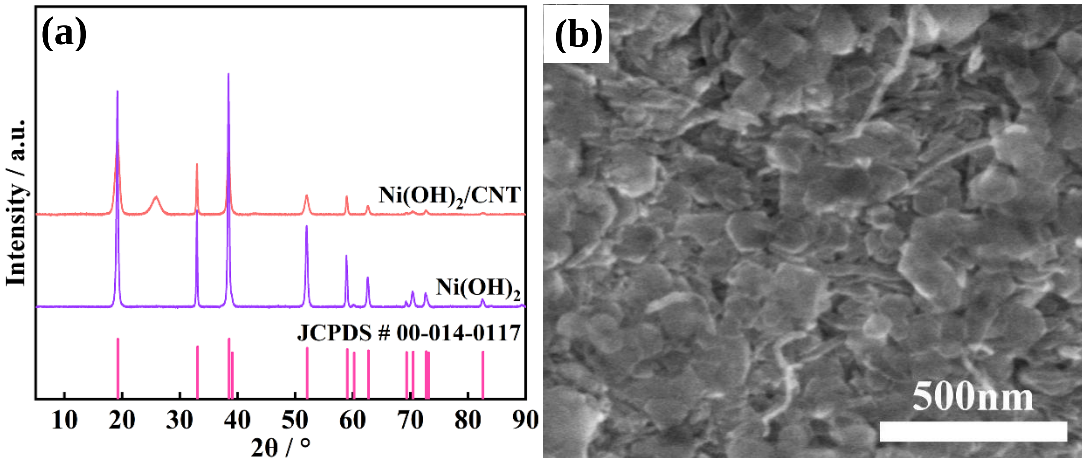


**Figure S1.** (**a**) XRD patterns of $Ni(OH)_2$/CNT and $Ni(OH)_2$; (**b**) SEM image of $Ni(OH)_2$/CNT.

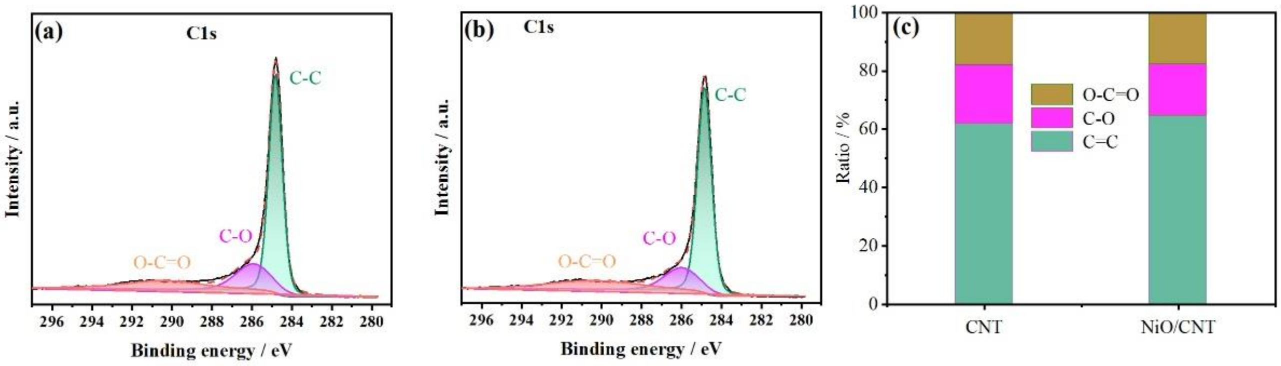


**Figure S2.** The C1s XPS spectra of CNT (**a**); NiO/CNT (**b**); the ratios of C element at different valence states (**c**).

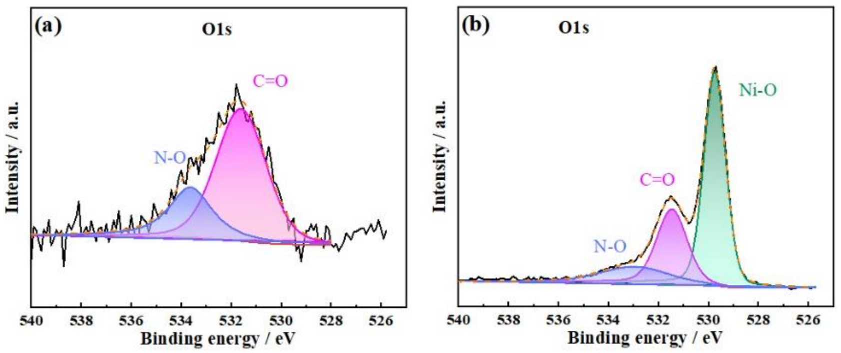


**Figure S3.** (**a**) The O1s XPS spectra of CNT and (**b**) NiO/CNT.

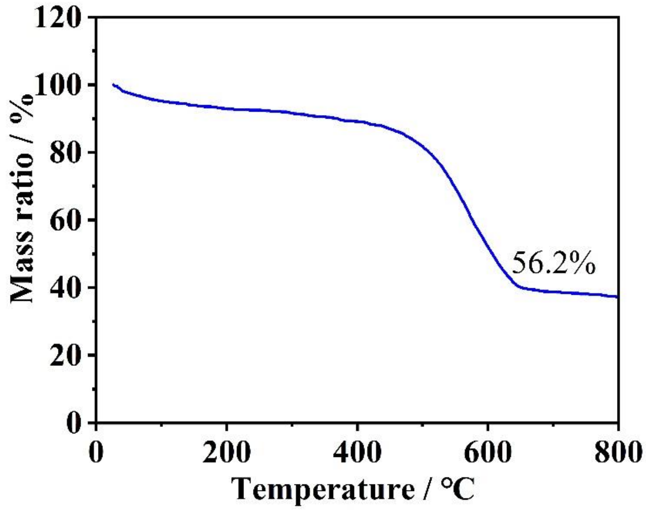


**Figure S4.** The TG curve of NiO/CNT catalysts.

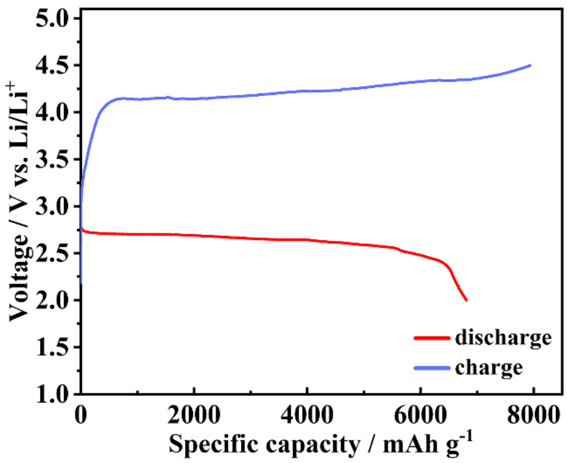


**Figure S5.** Discharge/charge curve at first cycle of Li-$O_2$ batteries with NiO/CNT. The NiO/CNT-mech electrode sheets are obtained by mixing CNT, NiO and PVDF with NMP solvent via ball-milling for 3h.

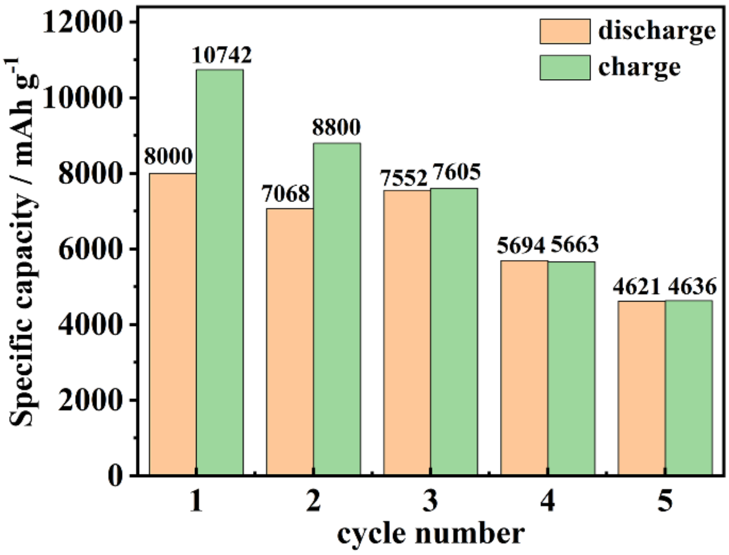


**Figure S6.** Five cycles of charge-discharge specific capacity comparison histogram of Li-$O_2$ battery with NiO/CNT cathode.

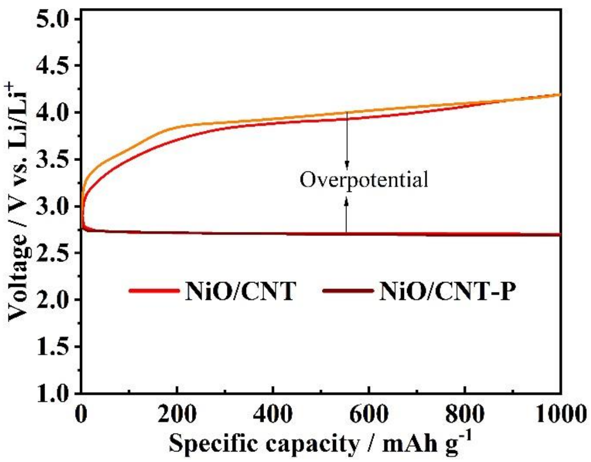


**Figure S7.** The charge and discharge curves of Li-$O_2$ batteries with NiO/CNT and NiO/CNT-P catalysts.

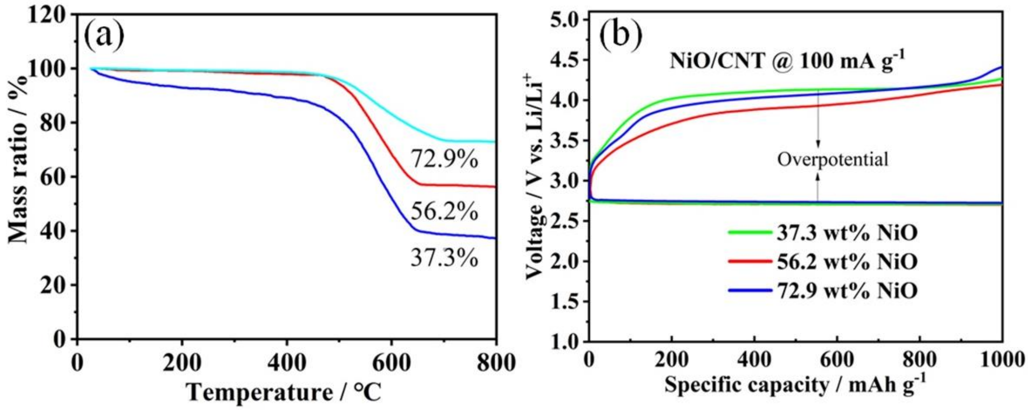


**Figure S8.** (**a**) The TG curve of NiO/CNT catalysts with different NiO contents ($O_2$ atmosphere, 10 °C $min^{-1}$); (**b**) the discharge and charge curves of Li-$O_2$ battery with NiO/CNT catalysts at a current density of 100 mA $g^{-1}$.

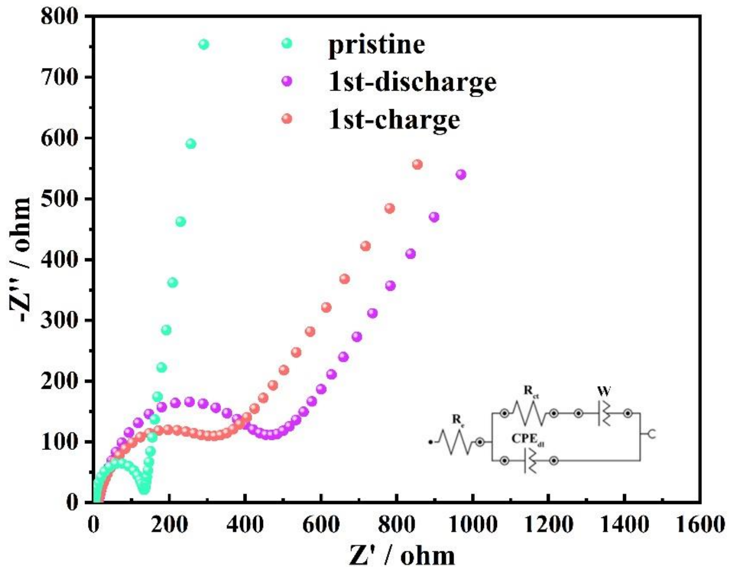


**Figure S9.** EIS of pristine Li-$O_2$ battery (blue), Li-$O_2$ battery for the 1st-discharge (purple), Li-$O_2$ battery for the 1st-charge (red).

**Table S1.** Parameters for the Li-$O_2$ battery with the NiO/CNT catalyst, fitted by the equivalent circuit in the inset.

| | $R_S$ (Ω) | $R_{ct}$(Ω) |
|---|---|---|
| Pristine | 7.7 | 84 |
| 1st discharge | 9.36 | 414 |
| 1st charge | 18.7 | 296 |

**Table S2.** Parameters for Li-$O_2$ battery with the CNT catalyst, fitted by the equivalent circuit in the inset.

| | $R_S$ (Ω) | $R_{ct}$(Ω) |
|---|---|---|
| Pristine | 7.13 | 111 |
| 1st discharge | 12.3 | 430 |
| 1st charge | 10.3 | 310 |

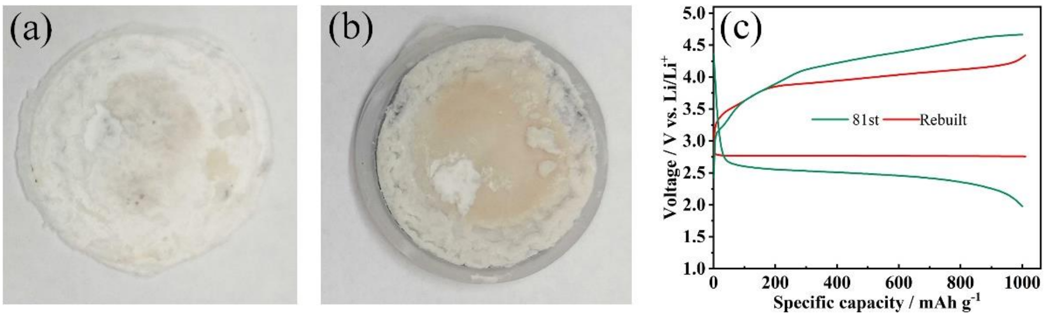


**Figure S10.** The photographs of (**a**) glass fiber and (**b**) lithium anode of Li-$O_2$ battery after 81 cycles with the NiO/CNT catalyst; (**c**) The discharge-charge profiles between the battery at 81st cycle and the rebuilt battery with the cycled NiO/CNT cathode, the fresh lithium foil, glassfiber and electrolyte.
